# Annual Variations and Influencing Factors of Zooplankton Community Structure in the Coastal Waters of Northern Shandong Peninsula, China

**DOI:** 10.3390/biology14101386

**Published:** 2025-10-11

**Authors:** Xiuxia Wang, Mingming Zhu, Bingqing Xu, Yanyan Yang, Xiaomin Zhang, Shaowen Li, Tiantian Wang, Fan Li, Guangxin Cui, Xiang Zheng

**Affiliations:** 1 Shandong Marine Resource and Environment Research Institute, Observation and Research Station of Laizhou Bay Marine Ecosystem, Yantai 264006, China; xiuxia.888@163.com (X.W.); ytsze9@163.com (M.Z.); xbq6688482@163.com (B.X.); xqdlmu@163.com (Y.Y.); zhangxiaominyt@163.com (X.Z.); lswyt2002@163.com (S.L.); xinlgc@163.com (G.C.); z544511346@163.com (X.Z.); 2 Yantai Marine Economic Research Institute, Yantai 264000, China; wangtt2009@163.com

**Keywords:** zooplankton, community structure, spatiotemporal distribution, annual variation, environmental factors, ontogenetic shifts

## Abstract

**Simple Summary:**

As an important food organism for fish, zooplankton is of great significance to ecological environment and evaluation of spawning grounds. However, the depth and systematic research on the variation patterns and influencing factors of zooplankton communities in the spawning grounds of the northern Shandong Peninsula waters are poorly understood. In this study, we conducted field sampling and investigated the spatiotemporal distribution of zooplankton community structure and the major environmental factors to explore the principles underlying their effects on the zooplankton community. The results showed that the abundance and biomass of zooplankton in the surveyed area exhibited significant annual variations, both showing a trend of first decreasing and then increasing, and nearshore higher than offshore. The overall low level of community diversity and its significant annual variations indicated that the zooplankton community structure in the surveyed area was unstable and showed a trend of degenerative succession. The community structure of zooplankton and larger-bodied dominant species showed stronger correlations with phytoplankton dynamics, whereas smaller-bodied species were more influenced by water temperature. Our study can provide references for habitat assessment of spawning grounds, as well as the protection and restoration of marine biodiversity in the coastal waters of the northern Shandong Peninsula.

**Abstract:**

The coastal waters of the northern Shandong Peninsula have abundant fishery resources, which serve as a critical transitional fishing ground for economic fish migrating into the Bohai Sea for spawning and departing for overwintering habitats. However, anthropogenic pressures such as garbage dumping have led to severe degradation of local fishery resources and concomitant adverse effects on zooplankton communities. To assess these impacts, we analyzed the spatiotemporal distribution, community structure, dominant species, and diversity indices of zooplankton based on sampling data collected in spring from 2015 to 2018 in this region. A total of 24 zooplankton species and 11 larval classes were identified, with the highest species richness observed in 2016. *Calanus sinicus* and *Centropages abdominalis* were the primary dominant species, with *C. sinicus* consistently predominant across all four years. Notably, the dominant species exhibited marked annual variability. The abundance and biomass of zooplankton in the surveyed area exhibited significant annual variations, both showing a trend of first decreasing and then increasing. Peak abundance occurred in 2015 (594.36 ind/m^3^), while the lowest was recorded in 2017 (118.73 ind/m^3^). Spatially, abundance and biomass were heterogeneous, with coastal waters exhibiting higher concentrations than offshore areas. The overall low level of community diversity and its significant annual variations indicated that the zooplankton community structure in the surveyed sea area was unstable and showed a trend of degenerative succession. The community structure of zooplankton and larger-bodied dominant species showed stronger correlations with phytoplankton dynamics, whereas smaller-bodied species were more influenced by water temperature.

## 1. Introduction

Zooplankton represents a critical trophic link in marine ecosystems, functioning as a key regulatory functional group that mediates energy transfer and matter cycling [1]. As the primary forage resource for higher trophic levels, shifts in zooplankton community structure directly govern the efficiency and direction of energy flow and material exchange within marine food webs. Empirical studies demonstrate that zooplankton aggregations serve as optimal foraging grounds for larval and juvenile fish, with species such as *Engraulis japonicus*, *Thrissa kammalensis* and *Alloisochaetus chinensis* exhibiting distribution patterns strongly correlated with zooplankton biomass [2,3]. Furthermore, the habitat quality of spawning grounds—often modulated by zooplankton dynamics—profoundly influences the survival and development of fish early life stages, thereby determining recruitment success in marine fish populations [4,5]. Consequently, investigating zooplankton community composition and spatiotemporal distribution is essential for (i) assessing ecosystem health, (ii) identifying critical spawning and nursery habitats, and (iii) informing sustainable fisheries management strategies.

The northern coastal waters of the Shandong Peninsula constitute a semi-enclosed, warm-temperate marine ecosystem encompassing several bays, including Taozi Bay, Shuangdao Bay, and Weihai Bay. This area has rich biological resources and serves three critical ecological functions: as a key passage for the reproductive migration of economically important fish species into the Bohai Sea and the subsequent out-migration of larval fish, such as *Scomber japonicus*, with peak spawning activity occurring in spring [6]. In 2015 and 2016 surveys, 59 and 48 species of fishery resources were caught in this area, respectively [7]. Given these ecological roles, research on spawning habitat dynamics in this region is of paramount importance for both conservation and fisheries management.

Recent years have seen a growing body of research on the spatiotemporal dynamics of zooplankton diversity indices, community structure, and their seasonal variations, as well as zooplankton responses to ocean currents and water masses. Zuo et al. [8], for example, classified zooplankton communities in the continental shelf regions of the East Sea and Yellow Sea during spring and autumn. Yang et al. [9] and Zou et al. [10] investigated zooplankton community structure and seasonal variations in the North Yellow Sea. Also, the relationship between zooplankton community structure and environmental factors in the Sishili Bay was examined [11]. Wang Rong et al. [12] conducted a preliminary analysis of zooplankton as ecological indicators in the Yellow Sea Warm Current. However, research on the annual variability of zooplankton community structure and its response to current systems in the northern coastal waters of the Shandong Peninsula remains limited. The purpose of this study is to explore the interannual variation patterns of the community structure of zooplankton during the main spawning period in the Yanwei Fishing Ground and analyze its influencing factors, hoping to provide data support for the assessment of feed organisms in the spawning ground. We hypothesize that the zooplankton community structure in this region experiences significant annual changes. These changes are mainly influenced by variations in factors such as phytoplankton abundance and water temperature. To address this gap, this study analyzes zooplankton monitoring data collected during spring (2015–2018) from the northern coastal waters of the Shandong Peninsula, with the following objectives: (i) characterize zooplankton community structure and its annual variability; (ii) evaluate the influence of environmental factors on zooplankton assemblages. The results of this study can provide references for habitat assessment of spawning grounds, as well as the protection and restoration of marine biodiversity in the coastal waters of the northern Shandong Peninsula.

## 2. Materials and Methods

### 2.1. Study Area and Stations

**Figure 1:**
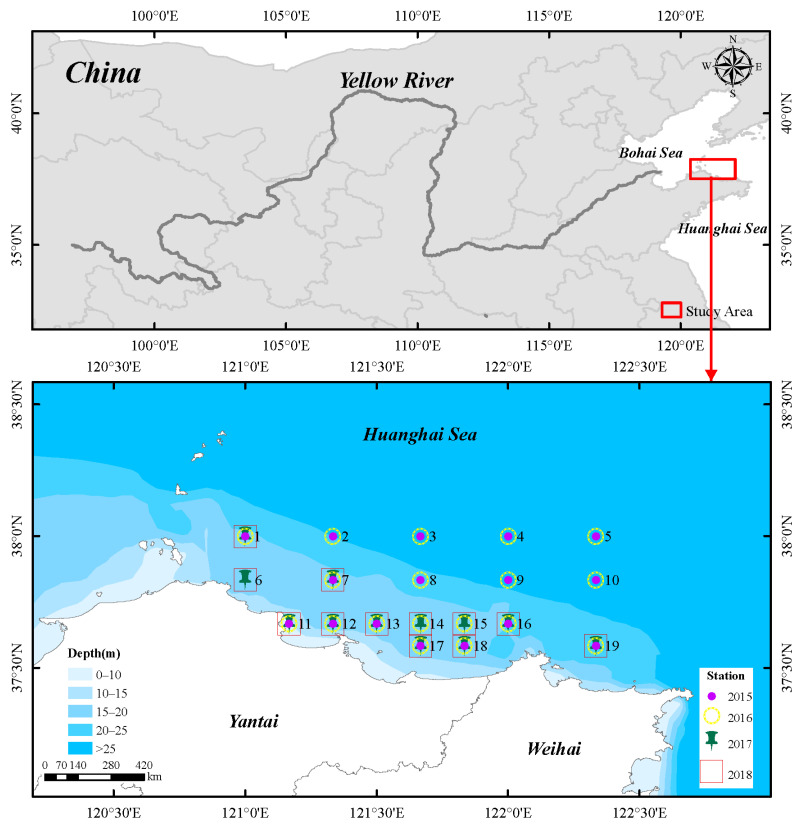
Sampling stations in the coastal waters along the northern Shandong Peninsula.

The study area was situated in the North Yellow Sea (35°35′–38°00″ N, 120°00′–122°20′ E), characterized by shallow waters with depths down to 40 m (Figure 1). From 2015 to 2018, four field surveys were conducted in spring, with 16, 18, 12 and 12 stations set up in each survey, respectively.

### 2.2. Data Sources

#### 2.2.1. Biological Data

Zooplankton and phytoplankton samples were collected vertically from bottom to surface using two specialized plankton nets, respectively: a shallow sea Type I net (aperture: 505 μm, net length: 145 cm, mouth area: 0.2 m^2^) and a shallow sea Type III net (aperture: 77 μm, net length: 140 cm, mouth area: 0.1 m^2^). Immediately after collection, samples were preserved in a 5% formaldehyde solution and transported to the laboratory for classification and determination.

In the laboratory, zooplankton samples were identified and counted under a Zeiss Stemi 508 stereo-microscope, with abundance calculated as individuals per cubic meter of seawater (ind·m^−3^). Biomass (wet weight) was weighed using an electronic balance (Ohaus Corporation, Parsippany, NJ, USA, DV215CD). For phytoplankton samples, 25 mL was placed into a counting chamber, allowed to settle and precipitate for 24 h, and then identified and counted under an Olympus BX51 microscope (Olympus Corporation, Tokyo, Japan), and the counting results were expressed as cells per cubic meter (cells·m^−3^). The filtered water volume (unit: m^3^) was calculated based on the flow meter readings (HYDRO-BIOS Corporation, Kiel, German, 438111/E-Flow) and the net mouth area of the sampling net. All sampling and analytical procedures strictly adhered to the standardized protocols outlined in the “Specifications for Marine Surveys, Part 6: Marine Biological Survey” (GB/T12763.6-2007) [13].

#### 2.2.2. Environmental Factor Data

Sea surface temperature (SST) and salinity (SSS) data were derived from remote sensing sources. SST data were obtained from NASA’s MODIS Aqua and Terra global Level 3 thermal infrared product, featuring a monthly temporal resolution and a spatial resolution of 0.04° × 0.04° (4 km). These datasets were acquired from the Asia-Pacific Data Research Center (APDRC): http://apdrc.soest.hawaii.edu/, accessed on 10 July 2024. SSS data were sourced from the NASA Soil Moisture Active Passive (SMAP) satellite, with a monthly temporal resolution and a spatial resolution of 0.25° × 0.25° (25 km). The data had undergone atmospheric correction, sea surface salinity calibration and sea surface roughness correction. These data were obtained from the Physical Oceanography Distributed Active Archive Center (PODAAC): http://apdrc.soest.hawaii.edu/erddap/griddap, accessed on 10 July 2024.

### 2.3. Data Analysis

#### 2.3.1. Dominant Species

The biological properties analyzed included total abundance (*A*), biomass (*B*), species richness (*S*), the dominance index (*Y*), and the alternative rate of dominant species (*R*), calculated as follows:

(1) $$\mathrm{Dominance index}:Y=\frac{n_{i}}{N}f_{i}$$

(2) $$\mathrm{Alternative rate of dominant species}:R=\frac{a+b-2c}{a+b-c}\times 100\%$$

where *n_i_* represents the total abundance of the *i*th species across all sampling stations; *N* denotes the total abundance of individuals at all stations; and *f_i_* signifies the frequency of occurrence of the *i*th species. Species were classified as dominant when *Y* > 0.02 [14]. For temporal comparisons, a and b represent the number of dominant species in two adjacent sampling periods, respectively; while c denotes the number of dominant species common to both periods [15].

#### 2.3.2. Biodiversity

To quantify biodiversity, the following indices were calculated: the Marglef species richness index (*D*), the Shannon–Wiener diversity index (*H*′), and the Pielou uniformity index (*J*′), using the formulas below:

Marglef species richness index (*D*): *D* = (*S* − 1)/log_2_*N* (3)

(4) $$\mathrm{Shannon–Wiener diversity index}\;(H^{\prime }):H^{\prime }=\sum_{i=1}^{S}Pi\log_{2}Pi$$

Pielou uniformity index (*J*′): *J*′ = *H*′/log_2_*S* (5)

In the above equations, *S* represents the number of zooplankton species per sample; *N* denotes the total zooplankton abundance; *Pi* signifies the proportion of individuals belonging to the *i*th species relative to *N*. Zooplankton community composition was analyzed using Analysis of Similarity (ANOSIM) in the PRIMER 6.0 software package [16,17].

#### 2.3.3. Correlation of Environmental Factors

The relationship between zooplankton dominant species and environmental factors was analyzed using redundancy analysis (RDA) in Canoco 5.0. Environmental variables were analyzed without transformation, while the abundance data of zooplankton and phytoplankton were converted using log (x + 1). In the RDA ordination diagram, environmental factors were represented as vectors (arrows). Vector length indicates the strength of the relationship between each environmental factor and zooplankton distribution patterns. The cosine of the angle between a vector and an ordination axis reflects their correlation, with acute angles denoting positive relationships and obtuse angles indicating negative relationships. The correlation coefficient between each environmental factor and ordination axis quantifies their linear association [18].

Pearson correlation coefficients between zooplankton community metrics and environmental variables were computed using the psych package (v2.2.9) in R (v4.2.1; https://www.r-project.org/, accessed on 23 July 2024). Figures were drawn using Origin 2022 (OriginLab Corporation, Northampton, MA, USA), ArcGIS 10.6 (Esri, Corporation, Redlands, CA, USA), and Surfer 14 (Golden Software Corporation, Golden, CO, USA). Statistical significance was defined as *p* < 0.05 for all analyses.

## 3. Results

### 3.1. Species Composition and Dominant Species of Zooplankton

A total of 35 zooplankton taxa were recorded, comprising 24 identified species and 11 planktonic larval forms (Table 1). Copepods dominated the assemblage with 12 species (34.29% of total taxa), followed by Cnidaria (5 species, 14.29%). Single representatives were documented from Protozoa, Cladocera, Amphipoda, Decapoda, Euphausiacea, Chaetognatha and Tunicata. Nine taxa demonstrated annual persistence, consistently occurring in spring surveys from 2015 to 2018: the Copepods *Calanus sinicus*, *Paracalanus parvus*, *Centropages abdominalis*, *Oithona similis*, the Amphipoda *Themisto gracilipes*, the Chaetognatha *Sagitta crassa*, and larval stages of Bivalvia, Macrura (mysis) and Brachyura (zoea).

A total of eight species reached the dominant species status, mostly copepods (Table 2). *C. sinicus* maintained dominance throughout all four years, and exhibited the highest dominance index from 2015 to 2017. *C. abdominalis* was also a dominant species during 2015–2017. Three species showed intermittent dominance: *O. similis* (dominant in 2017), *S. crassa* (2015), and Macrura mysis larvae (2018). The alternative rate of dominant species (R) increased progressively from 60% (2015 to 2016) to 66.67% (2016 to 2017) and 83.33% (2017 to 2018). The most significant community shift occurred between 2017 and 2018, when only *C. sinicus* persisted as a common dominant species.

### 3.2. Annual Variation in Zooplankton

The lowest number of zooplankton species recorded was 15, observed in 2015. In contrast, the highest number, 24 species, occurred in 2016. One-way ANOVA revealed a significant difference in species richness across the years (*F*_(3,54)_ = 11.647, *p* < 0.01). Post hoc analysis indicated that species richness in 2016 was significantly higher than in both 2015 and 2018 (Table 3).

Zooplankton abundance decreased from 2015 to 2017 and increased from 2017 to 2018, with the lowest value recorded in 2017 and the highest in 2015. A one-way ANOVA indicated the abundance of zooplankton in 2015 was significantly different from that in 2016 and 2017 (*F*_(3,54)_ = 3.632, *p* < 0.05). Biomass followed a similar trend, declining to a minimum of 72.08 mg·m^−3^ in 2017. Post hoc analysis revealed the 2017 biomass was significantly lower than that recorded in 2015 (*F*_(3,54)_ = 3.455, *p* < 0.05).

### 3.3. Horizontal Distribution of Zooplankton

**Figure 2:**
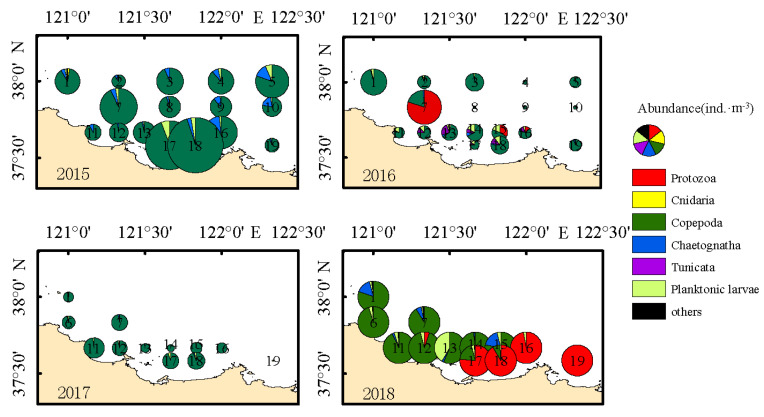
Spatial distribution of zooplankton abundance in the coastal waters along the northern Shandong Peninsula during spring from 2015 to 2018.

The results showed that the abundance of zooplankton in the surveyed area was generally higher nearshore than offshore, with obvious regional distribution (Figure 2). Copepods dominated the zooplankton community in 2015, with the highest abundance (2112.94 ind.m^−3^) in station 18. In 2016, *Noctiluca scintillans* was the predominant species. The density center shifted to Station 7 (total abundance: 829.19 ind·m^−3^), with *N. scintillans* contributing 659.46 ind·m^−3^. Zooplankton distribution was relatively uniform but characterized by low overall abundance in 2017. The distribution trend in 2018 was similar to that in 2015, with higher abundances in the eastern area. Stations 16 and 19 exhibited notably high densities dominated by *N. scintillans*.

**Figure 3:**
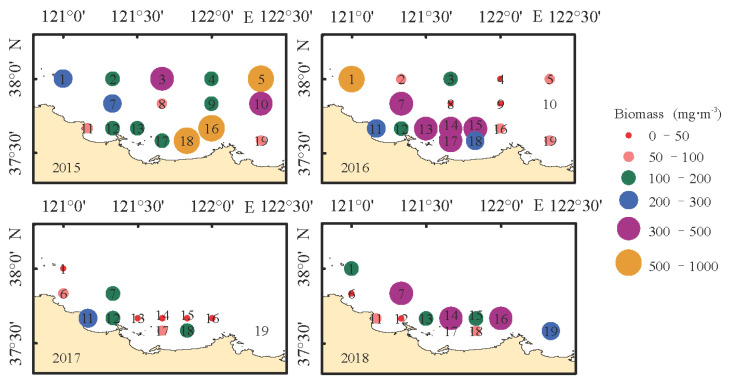
Spatial distribution of zooplankton biomass in the coastal waters along the northern Shandong Peninsula during spring from 2015 to 2018.

The horizontal distribution trend of zooplankton biomass was consistent with the abundance patterns, showing consistently higher values nearshore than offshore (Figure 3). Biomass also exhibited significant annual variation, peaking in 2015 and reaching its lowest level in 2017.

### 3.4. Community Structure Analysis

Significant annual variation occurred in zooplankton diversity index (*H*′) within the study area (*F*_(3,54)_ = 10.540, *p* < 0.01). Values peaked in 2016 (range: 0.72–2.43) and were lowest in 2018 (range: 0.08–1.53).

Species uniformity index (*J*′) was consistently low (mean = 0.47) with a small range of variation, yet showed significant differences among years (*F*_(3,54)_ = 6.943, *p* < 0.01).

**Figure 4:**
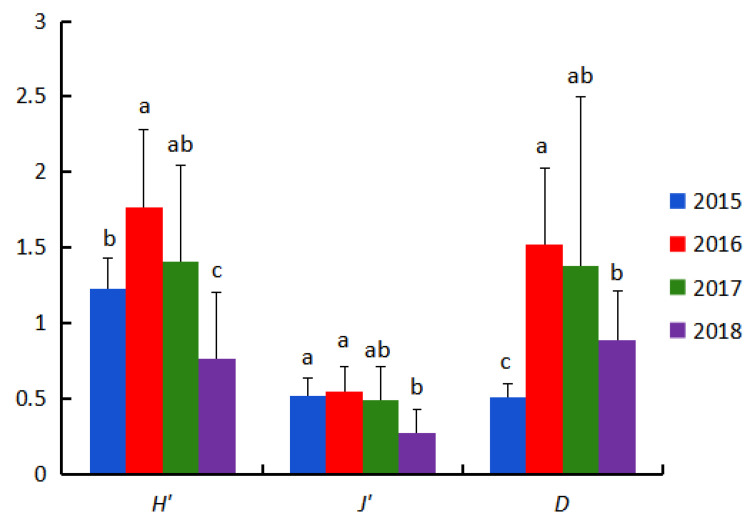
Annual variations in diversity indices of zooplankton community in the coastal waters along the northern Shandong Peninsula during spring from 2015 to 2018.

Species richness index (*D*) was significantly higher in 2016 and 2017 than in other years (*F*_(3,54)_ = 8.781, *p* < 0.01), reaching its lowest value in 2015 (Figure 4).

### 3.5. Analysis of Environmental Parameter

**Figure 5:**
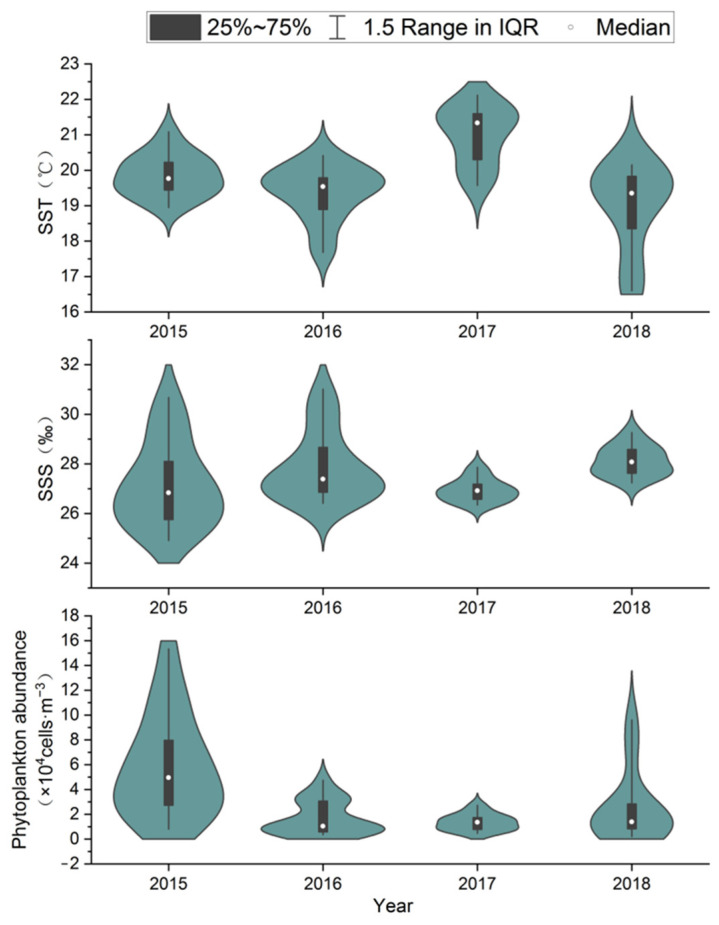
Annual variations in water temperature, salinity and phytoplankton abundance in the coastal waters along the northern Shandong Peninsula during spring from 2015 to 2018.

During spring surveys from 2015 to 2018, seawater temperature in the northern Shandong Peninsula ranged from 16.60 °C to 22.12 °C, with most stations recording 19–21 °C (Figure 5). Temperature in 2017 was significantly higher (mean = 21.01 °C) than in other years (*F*_(3,54)_ = 13.198, *p* < 0.01). Due to larger geographical coverage, salinity exhibited greater variation during 2015–2016 surveys (24.91–31.02‰). In contrast, the nearshore-focused 2017–2018 surveys showed reduced variability (26.44–29.27‰). Salinity was significantly lower in 2017 than in 2018 (*F*_(3,54)_ = 2.799, *p* < 0.05), but showed no significant differences between other survey years. A total of 25, 38, 20, and 20 phytoplankton species were identified in May of 2015, 2016, 2017, and 2018, respectively. The communities were predominantly composed of diatoms (23, 33, 17, and 16 species), followed by dinoflagellates (1, 4, 3, and 3 species). A small number of species belonging to other phyla were also recorded. Phytoplankton abundance showed a trend of decreasing first and then increasing (range: 0.80–15.34 × 10^4^ cells/m^3^), with 2015 values differing significantly from all other years (*F*_(3,54)_ = 9.303, *p* < 0.01).

**Figure 6:**
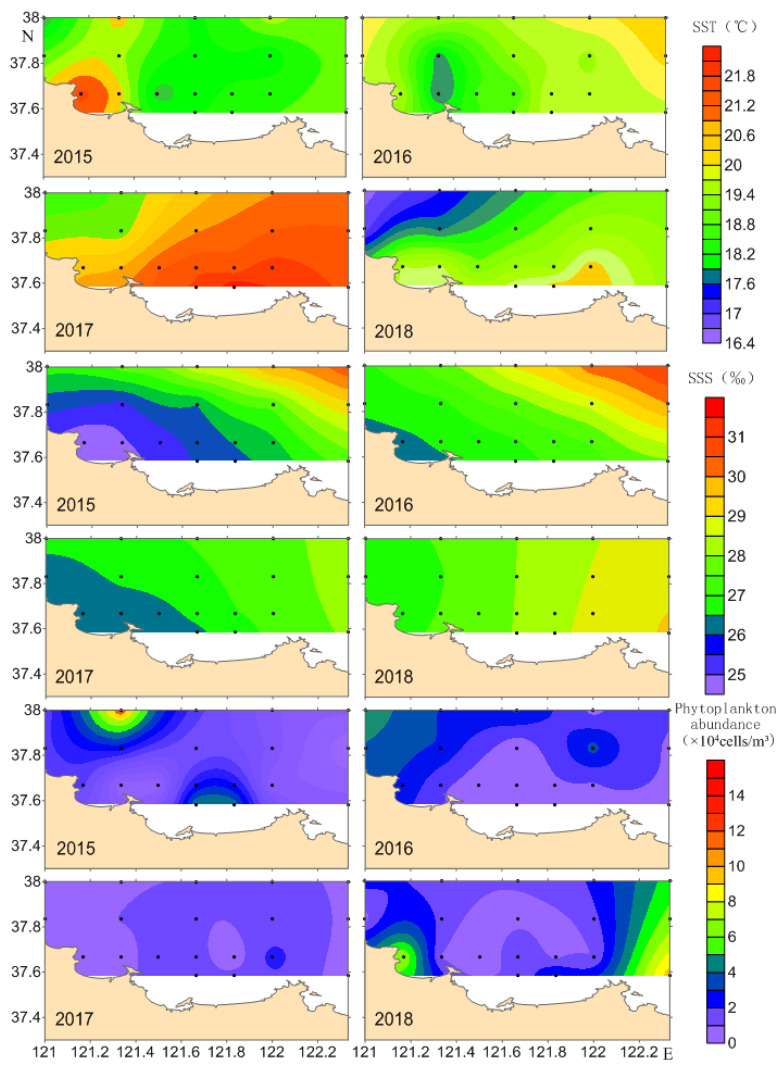
Temporal and spatial variations in temperature, salinity and phytoplankton abundance in the coastal waters along the northern Shandong Peninsula during spring from 2015 to 2018.

Sea surface temperature exhibited significant spatial heterogeneity (Figure 6). In 2015, temperature peaked near Sishiliwan Bay, whereas 2016 depressed values in this region. During 2017–2018, the trend of temperature distribution in the surveyed sea areas were consistent, with a rising from northwest to southeast, though 2017 maintained higher overall temperature. Salinity formed a distinct northeastward-increasing gradient from coastal southwest waters, with notable minima near Sishiliwan Bay. Phytoplankton abundance displayed marked patchiness. In 2015, phytoplankton abundance at station two represented an extreme outlier (15.34 × 10^4^ cells/m^3^), while other stations were all below 10 × 10^4^ cells/m^3^.

### 3.6. Relationships Between Zooplankton and Environmental Factors

**Figure 7:**
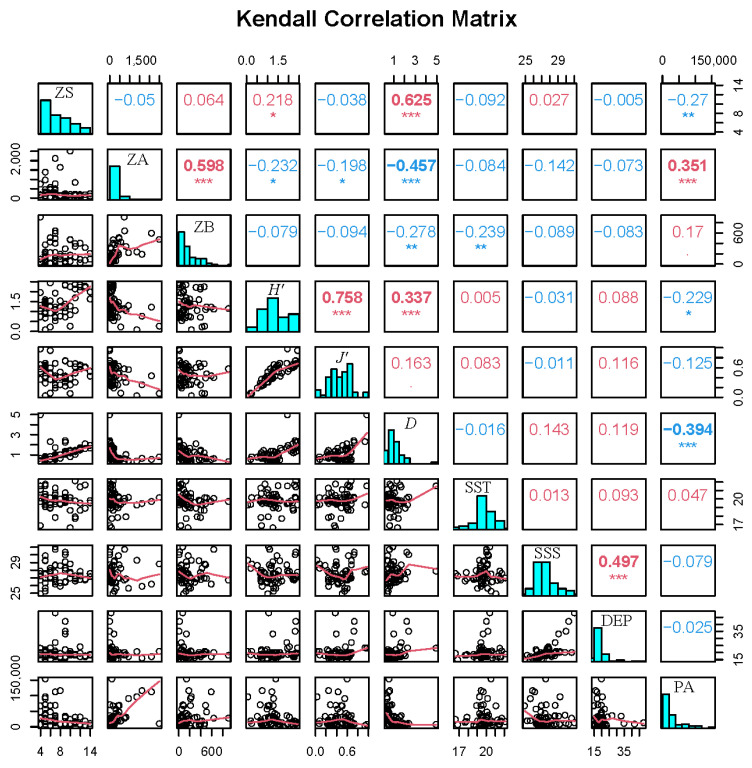
Pearson correlation matrices between zooplankton structure community and environmental factors, *: *p* < 0.05, **: *p* < 0.01, ***: *p* < 0.001. The histogram shows the frequency distribution of factors, black circles indicate bivariate plots, and red lines indicate smoothing curves for bivariate plots. ZS = zooplankton species, ZA = zooplankton abundance, ZB = zooplankton biomass, SST = sea surface temperature, SSS = sea surface salinity, DEP = depth, PA = phytoplankton abundance.

Analysis of correlations between zooplankton community structure and environmental factors revealed phytoplankton abundance as the strongest predictor (Figure 7). Zooplankton abundance showed a significant positive correlation with phytoplankton abundance (r = 0.351, *p* < 0.001). However, the number of species, diversity index, and richness index were all significantly negatively correlated with phytoplankton abundance (*p* < 0.05). Correlations with salinity and water depth were comparatively weaker. Zooplankton biomass exhibited a significant negative correlation with water temperature (r = −0.239, *p* < 0.01).

Redundancy analysis (RDA) revealed that the first two axes explained a cumulative 77.03% of the variation in the relationship between dominant zooplankton species and environmental factors (Table 4). The first axis (eigenvalue = 0.1170) had a species–environment correlation of 0.6175, while the second axis (eigenvalue = 0.0564) had a correlation of 0.5080. Both the first axis and the overall model (all axes) were statistically significant (*p* < 0.05). The VIF values for water temperature, salinity, water depth, and phytoplankton abundance were 1.033, 1.884, 1.898, and 1.033, respectively, indicating no multicollinearity.

**Figure 8:**
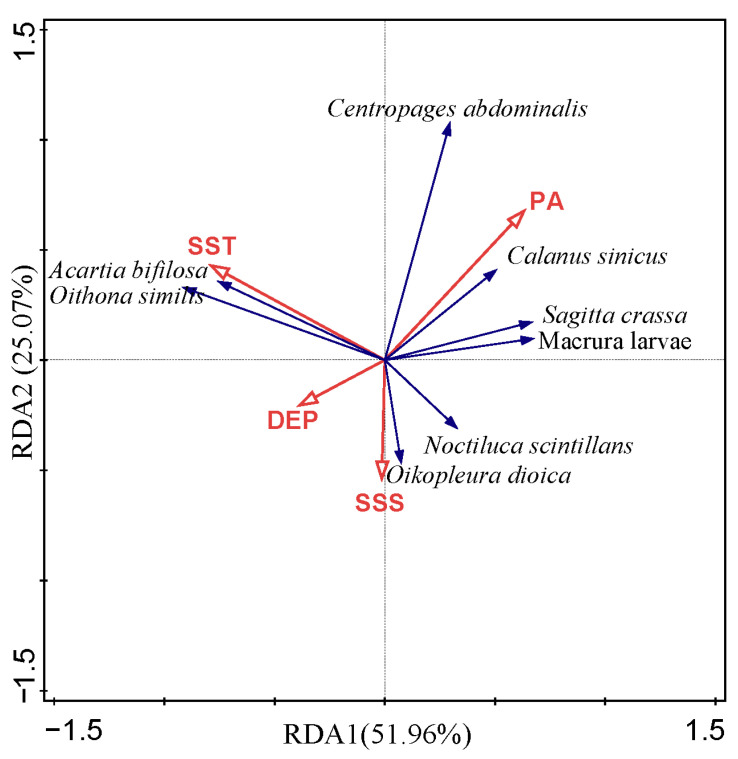
RDA ranking of dominant zooplankton species with environmental factors.

Dominant species–environment relationships (Figure 8) showed distinct groupings: Large-bodied species were associated with shallow water and high phytoplankton abundance, such as *C. sinicus*, *C. abdominalis*, and *S. crassa*. Small-bodied species (e.g., *Acartia bifilosa*, *O. similis*) were associated with higher water temperatures and lower phytoplankton abundance. *N. scintillans* and *Oikopleura dioica* were associated with low water temperature, low phytoplankton abundance, and high salinity.

## 4. Discussion

### 4.1. Species Composition

According to the research of Tang and Ye [19], the northern waters of Shandong Peninsula in spring were mainly influenced by two water masses: the Yellow Sea Cold Water Mass (YSCWM) and the Bohai-Laizhou Coastal Current (BLCC). By May, the Yellow Sea Cold Water Mass occupies the coast of Yantai and Weihai in the northern Yellow Sea, with a temperature of around 5 °C. At this time, the Bohai-Laizhou Coastal Current flows northward along the Bohai coast and extends into the study area through the southern end of the Bohai Strait. The interaction of these systems establishes a pronounced cross-shelf salinity gradient, increasing from nearshore to offshore. This hydrographic framework aligns with our observed zooplankton distribution: community composition was dominated by neritic low-salinity species in nearshore zones, and the dominant species were mostly low-salinity or euryhaline warm-water taxa. These patterns corroborate established findings that zooplankton serve as biological indicators for currents [20,21] and water mass [22] distributions.

A total of 25 species of zooplankton and 10 types of planktonic larvae were recorded in this study, with a higher proportion of low-salinity nearshore species and eurythermal/euryhaline species. This composition aligns broadly with reported zooplankton assemblages in the Yellow and Bohai Seas [23]. Annual variation in species composition was insignificant, indicating overall stability. Species richness peaked in 2016, while 2017 and 2018 exhibited lower but comparable diversity. In contrast, 2015 recorded the fewest species yet the highest zooplankton abundance. This anomaly may be attributed to elevated precipitation in 2015, which reduced salinity and increased phytoplankton biomass—conditions favoring proliferation of copepods such as *C. sinicus* and *C. abdominalis*.

Previous studies confirm that water temperature and salinity were pivotal drivers of zooplankton spatiotemporal distribution, with their spatial gradients determining dominant species patterns [24,25,26]. As a major group of zooplankton, copepods were the dominant species in all four years of the study, particularly *C. sinicus*, which provides forage for various economically important fish species and was the absolutely dominant species across all four years. This is mainly because *C. sinicus* belongs to a eurythermic–euryhaline species, which confers strong adaptability to environmental variability [27]. Furthermore, significant declines in populations of planktivorous fish (e.g., *Larimichthys polyactis* and *Scomber japonicus*) occurred due to persistent overfishing and pollution in the study area [28,29]. This trophic release likely enhanced *C. sinicus* growth and reproduction by reducing predation pressure.

Except for *C. sinicus*, the temperate coastal species *C. abdominalis* remained dominant from 2015 to 2017. In contrast, traditional dominants such as *S. crassa* exhibited declining prominence. This shift reflects plankton community regression and signals degradation of fishery resources in the study area.

### 4.2. Horizontal Distribution and Annual Variation in Zooplankton

Previous research has shown that the horizontal distribution of zooplankton was influenced by environmental factors such as water temperature, salinity, and chlorophyll *a*, which in turn affected the quantity and distribution of fish species that feed on zooplankton in the upper and middle water layers [30]. In this study, both the biomass and abundance of zooplankton were positively correlated with phytoplankton abundance, with the strongest correlation, while they were negatively correlated with water temperature and salinity, aligning with the findings of Wang et al. [23]. The dense areas of zooplankton coincided with the dense areas of fish eggs and larvae, such as *Scomber japonicus* and *Engraulis japonicus* [6], indicating a close relationship between zooplankton and the distribution of spawning grounds.

Zooplankton abundance in this study area exhibited significant annual variability during spring. In 2015, despite the lowest species count, the abundance reached its peak, with the highest abundance values observed for the eurythermic and euryhaline species such as *C. sinicus*, as well as the coastal low-salinity species such as *C. abdominalis* and *S. crassa*. The possible reason was that the Bohai-Laizhou Coastal Current influenced the surveyed waters, forming a low-salinity environment in the nearshore area. At the same time, the Yellow Sea Cold Water Mass was relatively far from the shore, resulting in higher temperatures near the shore and lower temperatures farther away, thus providing low-salinity and warm temperature conditions in the nearshore areas that were suitable for the growth of the aforementioned species. Crucially, 2015 also recorded peak phytoplankton abundance—driven by optimal salinity/temperature conditions and the absence of *N. scintillans* blooms. This combination created an ideal trophic scenario: abundant prey resources without competition from red-tide organisms, synergistically enhancing zooplankton growth and reproduction. These conditions collectively drove the observed zooplankton abundance maximum.

The species of zooplankton remained relatively stable across the study period (2016–2018), whereas abundance exhibited significant annual variability, reaching its lowest level in 2017 and peaking in 2018. This pattern likely reflects the dominance of *N. scintillans* in both 2016 and 2018, which contributed substantially to total zooplankton biomass in these years. Previous studies have demonstrated that the distribution of *N. scintillans* was strongly influenced by environmental parameters, including nutrient concentrations (e.g., DIN, DIP), sea surface temperature, and dissolved oxygen (DO) levels [31]. The persistent dominance of *N. scintillans* in our study area suggests springtime eutrophication, a condition that may elevate the risk of algal blooms. Notably, eutrophic conditions can also promote proliferation of unpalatable phytoplankton (e.g., cyanobacteria and chlorophytes), thereby indirectly suppressing zooplankton through bottom-up control of food resources. Furthermore, shifts in fish foraging behavior—whereby planktivorous species increase zooplankton predation under declining edible algal availability—may have exacerbated zooplankton depletion [32]. Consistent with these mechanisms, our data revealed markedly lower zooplankton abundance in 2016 and 2018 after excluding *N. scintillans* from the analysis, supporting the hypothesis that eutrophication-driven trophic cascades negatively impacted non-*N. scintillans* zooplankton populations.

### 4.3. Biodiversity and Stability of Community Structure

The diversity index of zooplankton communities is strongly influenced by species richness, with higher species numbers typically corresponding to increased diversity [33,34]. However, pronounced dominance by one or a few species can significantly reduce diversity, as observed in previous studies [35,36]. In this study, zooplankton diversity was calculated based on abundance-weighted indices. The diversity index peaked in 2016, followed by a gradual decline, reaching its lowest value in 2018. Notably, while 2015 exhibited the lowest species richness, the strong dominance of key taxa such as *C. sinicus* and *C. abdominalis* contributed to reduced diversity. Similarly, in 2018, the overwhelming dominance of *N. scintillans* further suppressed diversity, resulting in the lowest recorded index. In contrast, 2016 displayed the highest number of dominant species, but with moderate individual dominance levels, allowing diversity to reach its maximum. Statistical analysis supported these observations, revealing a positive correlation (r = 0.218) between species richness and the diversity index in the study area. These findings align with established ecological principles regarding diversity–dominance relationships in marine plankton communities.

The species diversity index is a well-established metric for assessing the stability of community structure, often analyzed in conjunction with complementary indicators such as dominant species turnover rate [37,38]. In this study, zooplankton communities exhibited a consistently high and increasing dominant species replacement rate, coupled with a declining trend in species diversity. This inverse relationship suggests a progressive destabilization of the zooplankton community structure in the study area. Such structural instability may be attributed to anthropogenic pressures, such as environmental pollution. The results of the correlation analysis showed that the diversity index of zooplankton and the dominant species with larger individuals had a relatively strong correlation with phytoplankton. However, existing studies have indicated that the nutrient flux in this area was at a relatively low level [39], and activities such as garbage dumping have a significant impact on the marine environment, resulting in a relatively low diversity of phytoplankton [40]. Therefore, it is inferred that environmental pollution caused by human activities indirectly affects the community structure of zooplankton. These disturbances can disrupt trophic interactions, promote opportunistic species dominance, and ultimately reduce ecosystem resilience. The observed patterns align with documented cases of marine community degradation under sustained anthropogenic stress.

### 4.4. Limitation Analysis

Due to the limitations of the survey data, this study has several limitations. First, in order to retain more information, we kept all the survey data. The inconsistent number of sampling stations across different years may affect the accuracy of comparisons. However, we can compare the situations of the same survey stations through planar distribution maps. Second, this study relies on satellite-derived environmental data, which has a relatively low spatial resolution compared to sampling and may introduce certain errors in the analysis of the relationship between zooplankton community structure and environmental factors. We hope that by exploring the correlation between remote sensing data and biological data, we will be able to predict biological data through remote sensing data in the future, thereby reducing the disturbance to marine organisms caused by field surveys and reducing the cost of scientific research surveys. Third, although this study utilized redundancy analysis (RDA) to demonstrate the correlations between zooplankton, environmental factors, and phytoplankton, it cannot prove causality. We hope that there will be a deeper analysis in the future to validate this conclusion with more survey data.

## 5. Conclusions

This study investigated zooplankton community structure in the coastal waters of the northern Shandong Peninsula from 2015 to 2018. A total of 24 zooplankton species and 11 planktonic larval taxa were identified, with Copepoda representing the dominant group (followed by Cnidaria). Annual variability was pronounced in both species’ composition and abundance. The highest species occurred in 2016, while peak total abundance (including and excluding *N. scintillans*) was observed in 2015. Dominant species exhibited significant annual turnover, though *C. sinicus* remained persistently dominant throughout the study period. Community diversity remained consistently low with marked annual fluctuations, reflecting unstable community structure in the surveyed area. Correlation analyses revealed the following: zooplankton diversity and dominant species with larger individuals showed strong associations with phytoplankton, while the dominant species with smaller individuals correlated significantly with water temperature. The changes in zooplankton community structure were likely influenced by human activities.

The current research framework does not comprehensively reflect the complex relationships between zooplankton community structure and environmental drivers in the study area. We expect that there will be a deeper analysis in future studies. We hope that in the future, biological data can be predicted through remote sensing data, thereby reducing the disturbances of field surveys on marine organisms and reducing the cost of scientific research surveys. We also hope that this article can provide some references for fishery resource managers. The key to protecting marine biological resources is to reduce artificial damage, protect the marine environment, and provide a suitable habitat environment for marine organisms.

## Figures and Tables

**Table 1:** Species composition of zooplankton in the coastal waters along the northern Shandong Peninsula during spring from 2015 to 2018.

Groups	Species	Year			
		2015	2016	2017	2018
Protozoa	*Noctiluca scintillans*		√		√
Cnidaria	*Rathkea octopunctata*		√		√
	*Euphysora bigelowi*		√	√	
	*Clytia folleata*		√		
	*Proboscidactyla flavicirrata*		√		
	*Obelia* spp.	√			
Cladocera	*Evadne tergestina*		√	√	
Copepoda	*Calanus sinicus*	√	√	√	√
	*Paracalanus parvus*	√	√	√	√
	*Centropages abdominalis*	√	√	√	√
	*Eurytemora pacifica*			√	
	*Labidocera bipinnata*		√	√	
	*Acartia clausi*			√	
	*Acartia pacifica*	√			√
	*Acartia bifilosa*		√	√	√
	*Oithona similis*	√	√	√	√
	*Corycaeus affinis*		√	√	√
	*Oithona brevicornis*			√	
	*Microsetella norvegica*			√	√
Amphipoda	*Themisto gracilipes*	√	√	√	√
Decapoda	*Leptochela gracilis*	√			
Euphausiacea	*Euphausia pacifica*	√			
Chaetognatha	*Sagitta crassa*	√	√	√	√
Tunicata	*Oikopleura dioica*		√		√
Planktonic larvae	*Polychaeta larvae*		√		√
	*Gastropoda larvae*		√		√
	*Bivalvia larvae*	√	√	√	√
	*Copepoda nauplius larvae*		√	√	√
	*Gammarus* sp.		√		√
	*Macrura mysis larvae*	√	√	√	√
	*Macrura zoea larvae*	√			
	*Brachyura zoea larvae*	√	√	√	√
	*Ophiopluteus larvae*	√			√
	*Fish eggs*		√	√	√
	*Fish larvae*		√	√	√

Note: √ indicates the presence of corresponding species.

**Table 2:** Annual dominance variability of dominant zooplankton species in the coastal waters along the northern Shandong Peninsula.

	Dominance	Year			
Species		2015	2016	2017	2018
*Noctiluca scintillans*		-	0.068	-	0.301
*Calanus sinicus*		0.457	0.441	0.649	0.231
*Centropages abdominalis*		0.437	0.173	0.130	-
*Acartia bifilosa*		-	-	0.044	-
*Oithona similis*		-	-	0.069	-
*Sagitta crassa*		0.066	-	-	-
*Oikopleura dioica*		-	0.033	-	-
*Macrura mysis larvae*		-	-	-	0.022

**Table 3:** Variation in zooplankton species number, abundance and biomass in the coastal waters along the northern Shandong Peninsula during spring from 2015 to 2018.

**Year**	**Species**	**Abundance (ind./m^3^)**	**Biomass (mg/m^3^)**
2015	5.44 ± 1.17 ^b^	594.36 ± 541.43 ^a^	290.53 ± 235.52 ^a^
2016	9.94 ± 2.44 ^a^	167.35 ± 194.85 ^b^	200.31 ± 163.59 ^ab^
2017	7.83 ± 2.76 ^ab^	118.73 ± 77.08 ^b^	72.08 ± 67.7 ^b^
2018	7.25 ± 2.05 ^ab^	464.59 ± 717.34 ^ab^	183.82 ± 155.85 ^ab^

Note: Different superscript letters indicate significant differences (*p* < 0.05).

**Table 4:** RDA results of dominant zooplankton species and environmental factors.

Axis	Eigenvalue	Pseudo-Canonical Correlation	Cumulative Variable Percentage (%)		Sum of All Eigenvalues	Sum of All Canonical Eigenvalues
			Explained Variation (Cumulative)	Explained Fitted Variation (Cumulative)		
Axis1	0.1170	0.6175	11.70	51.96	1.000	0.2250
Axis2	0.0564	0.5080	17.34	77.03		
Axis3	0.0331	0.5388	20.65	91.76		
Axis4	0.0185	0.4102	22.51	100.00		

## Data Availability

The data that support the findings of this study are available from the corresponding author or the first author upon reasonable request.
